# Evaluation of Metabolic Syndrome and Its Associated Risk Factors in Type 2 Diabetes: A Descriptive Cross-Sectional Study at the Komfo Anokye Teaching Hospital, Kumasi, Ghana

**DOI:** 10.1155/2019/4562904

**Published:** 2019-05-02

**Authors:** Francis Agyemang-Yeboah, Benjamin Ackon Jnr. Eghan, Max Efui Annani-Akollor, Eliezer Togbe, Sampson Donkor, Bright Oppong Afranie

**Affiliations:** ^1^Department of Molecular Medicine, School of Medical Sciences, Kwame Nkrumah University of Science and Technology (KNUST), Kumasi, Ghana; ^2^Medicine Department, School of Medical Sciences, Kwame Nkrumah University of Science and Technology (KNUST), Kumasi, Ghana; ^3^Department of Medical Laboratory Technology, Faculty of Allied Health Sciences, Kwame Nkrumah University of Science and Technology (KNUST), Kumasi, Ghana

## Abstract

*Background. *Metabolic syndrome (MS) is a collection of cardiovascular risk factors comprising insulin resistance, dyslipidemia, obesity, and hypertension, which may cause further complications in diabetes. Although metabolic syndrome (MS) is increasing in incidence in diabetics and leading to significant cardiovascular diseases and mortality, there is dearth of data in Ghana. This study investigated metabolic syndrome, its prevalence, and its associated risk factors in type 2 diabetes at the Komfo Anokye Teaching Hospital, Kumasi, Ghana.* Methods*. The study involved 405 diabetic patients attending the Diabetic Clinic of the Komfo Anokye Teaching Hospital (KATH) Kumasi, in the Ashanti Region of Ghana. A well-structured questionnaire was used to obtain demographic background such as their age and gender. Anthropometric measurements were obtained using the Body Composition Monitor (Omron ® 500, Germany) which generated digital results on a screen and also by manual methods. Fasting venous blood was collected for the measurement of biochemical parameters comprising fasting plasma glucose (FPG), glycated haemoglobin (HbA1c), high density lipoprotein cholesterol (HDL-c), and triglyceride (TG). Metabolic syndrome was defined according to the National Cholesterol Education Program Adult Treatment Panel III (NCEP ATP III).* Results. *Out of the total of 405 participants, 81 were males and 324 were females, and the estimated mean age was 58.5 ± 9.9 years. The female patients exhibited higher mean waist circumference (WC) and mean hip circumference (HC) as well as an approximately higher body mass index than males (28.3 ± 5.1, 26.5 ± 4.2 for the female and male respectively). Overall, the prevalence of metabolic syndrome observed among the study population was 90.6%.* Conclusions. *The prevalence of metabolic syndrome observed among the study population was 90.6%, with a higher percentage in females than males. High triglyceride levels and high waist circumference were the main risk factors for MS in the diabetic population.

## 1. Background

Metabolic syndrome (MS) is a combination of clinical and biological abnormalities which confers greater risk of type 2 diabetes (T2DM), cardiovascular disease (CVD) [1], and liver diseases [2]. A study in Ghana on the prevalence of MS in type 2 diabetes mellitus patients revealed an estimate of 58% [3]. The different parameters that comprise MS were initially described by Reaven in 1988 in what was called “syndrome X” [4]. These include abdominal obesity, higher-than-optimal plasma pressure, disorders of glucose metabolism, and abnormal lipid profile [4].

The underlying feature of all these abnormalities, though still debated, seems to be insulin resistance. Independent of any abnormalities of glucose metabolism, individuals with type 2 diabetes are at increased risk of MS [5, 6]. When diabetes mellitus and MS occur simultaneously the chances of cardiovascular risk and Chronic Kidney Disease (CKD) increase [6].

Prevalence studies of MS that have been conducted in West Africa, especially among Ghanaian diabetic patients, are few [7]. This study therefore evaluated metabolic syndrome in type 2 diabetes and the risk factors associated with it. It has been documented in several large epidemiology cohort studies that obesity and other indicators of metabolic syndrome are associated with cardiovascular outcomes in adults, such as cerebrovascular disease, myocardial infarction, and sudden death [8–10]. With the increasing mortality cases recorded among diabetics, identifying risk factors which predispose diabetic patients to MS may offer opportunities to modify lifestyle and develop therapeutic regimens where necessary to prevent further complications [11].

## 2. Methods

### 2.1. Study Design and Setting

This hospital-based cross-sectional study involved 405 diabetic patients attending the Diabetic Clinic of the Komfo Anokye Teaching Hospital (KATH) Kumasi, in the Ashanti Region of Ghana. It is located in Kumasi, the Regional capital of the Ashanti Region in Ghana with a total projected population of 4,780,380 (Ghana Statistical Service, 2010). KATH is a major Teaching Hospital and it is positioned in the middle belt of Ghana. It has over 1000 bed capacity. The relatively good road network and the cosmopolitan nature of Kumasi make the hospital accessible to all other areas.

### 2.2. Study Population

Using a simple random sampling technique, 405 patients visiting the diabetic clinic at KATH were recruited for the study. Using a prevalence of T2DM of 4% [12] in urban areas in Ghana, the Cochran's formula [13] was used to calculate the minimum population size required for this work and was 373 participants. However, to accommodate a nonresponse rate of 10% and stronger statistical power and effect size, the sample size was projected to 405.

Pregnant women, urinary tract infection (UTI) patients, and patients with other diagnosed chronic conditions apart from diabetes were excluded from this study. Written consent was obtained from all participants by explaining to them the aim of the project and their freedom to either participate or not, in their native language (Twi). Those literates read and voluntarily appended their signatures and the illiterates applied thumbprints to the consent forms. The study was approved by the Committees on Human Research Publication and Ethics (CHRPE) of the Kwame Nkrumah University of Science and Technology (KNUST) and the Komfo Anokye Teaching Hospital (KATH), Kumasi.

### 2.3. Questionnaire Administration and Data Collection

A well-structured questionnaire was administered to all participants by qualified research assistants. Items in the questionnaire included demographic background, educational level, socioeconomic status, and past and current drug history. Fasting venous blood was collected for the measurement of some biochemical parameters comprising fasting plasma glucose (FPG), high density lipoprotein cholesterol (HDL-c), and triglyceride using the Vitalab Flexor E (Vital Scientific BV, Spankeren/Dieren, The Netherlands) analyzer as well as glycated haemoglobin (HbA1c) using Variant II (Bio Rad, Hercules, CA, USA) analyzer.

### 2.4. Anthropometric Measurement

Anthropometric measurements were obtained using the Body Composition Monitor (Omron ® 500, Germany) which employs bioelectric impedance (BIA) and by manual methods. A Digital Stadiometer (seca 213) was used to measure height (cm). Furthermore, the waist and hip circumferences were each measured to the nearest 0.1cm by a tape measure, and the waist-to-hip ratio (WHR) was calculated as Wcm/Hcm. The Body Composition Monitor generated values for body fat percentage and calculated the BMI (Body Mass Index) from weight registered by the instrument and height (converted to metres) values manually entered.

### 2.5. Determination of Metabolic Syndrome

Metabolic syndrome was defined according to the modified National Cholesterol Education Program Adult Treatment Panel III (NCEP ATP III), since this classification has been proven to be more accurate [14, 15]. The modified NCEP, ATP III definition required at least three of the followings: (i) waist circumference (>90 cm in men and >80 cm in women for Asians), (ii) high triglyceride ≥150 mg/dl (1.7 mmol/l), (iii) low high density lipoprotein cholesterol (HDL-c) <40 mg/dl (1.03 mmol/l) in men and <50 mg/dl (1.29 mmol/l) in women, (iv) blood pressure (≥130/85 mmHg or current antihypertensive medication), and (v) fasting plasma glucose ≥100 mg/dl (≥5.6 mmol/l).

### 2.6. Statistical Analyses

Data was analyzed using GraphPad Prism version 5.00 and SPSS version 20 (SPSS Inc., Chicago, IL, USA). The results were expressed as mean ± SD. Unpaired t-test and one way analysis of variance (ANOVA) were used to compare mean values of continuous variables. Differences were considered to be statistically significant at p value less than 0.05. Metabolic syndrome was defined according to the National Cholesterol Education Program Adult Treatment Panel III (NCEP ATP III). The receiver operating characteristic (ROC) curve was used to establish the sensitivity, specificity, and the area under curve (AUC) to assess the accuracy and cut-off of selected indicators for MS.

## 3. Results

The estimated average age was 58.5 ± 9.9 years. Though the males were on average slightly younger than the females, the gender age difference was not statistically significant (p=0.554). Gender differences in the average levels of fasting plasma glucose FPG, percentage glycated haemoglobin (HbA1c (%)) and percentage individuals with HbA1c greater than 7 percent were not significant (p = 0.234, 0.643 and 0.551 respectively) (Table 1).

In general, the anthropometric indices of the male subjects in this study were found to be significantly different from their female counterparts. Waist-to-hip ratio (WHR) was the only anthropometric measurement that was not significant in terms of gender difference (p=0.924). All other measured parameters of weight, height, BMI, WC, and HC showed statistical differences when stratified by gender. The males exhibited higher means of weight and height than females. However, the females had higher means of BMI, WC, and HC than the males (Table 2).

**Figure 1:**
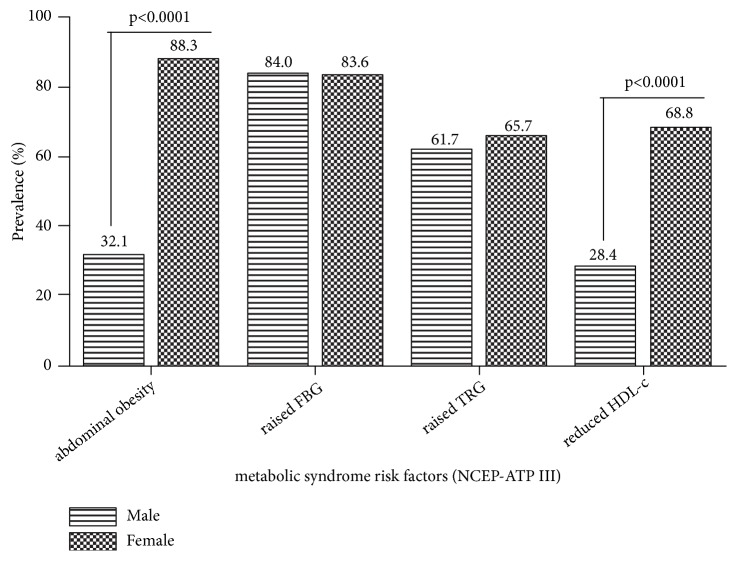
Prevalence of selected risk factors for metabolic syndrome stratified by gender.

It was observed from this study that female diabetic patients were more prone to presenting with abdominal obesity and reduced levels of high density lipoprotein cholesterol (HDL-c) than their male counterparts. However, gender differences in raised fasting plasma glucose and triglyceride were not statistically significant (Figure 1).

**Figure 2:**
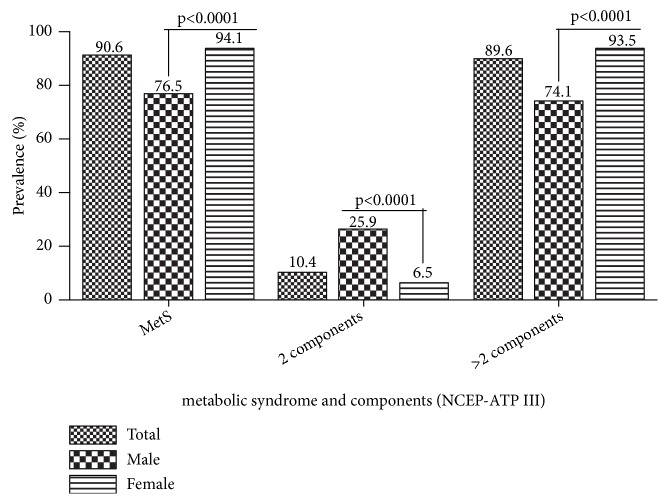
Prevalence of metabolic syndrome and its components among the study population with further stratification by gender.

In general the prevalence of metabolic syndrome observed among the study population was 90.6%. However, the MS condition among female participants (94.1%) was significantly higher than that of their male counterparts (76.5%) with p less than 0.0001. A significantly higher proportion of males exhibited two components of metabolic syndrome compared to the female. However, the reverse was observed with participants presenting with more than two components of metabolic syndrome, where the female proportion was significantly higher (Figure 2).

It was observed that all the selected demographic, biochemical, and anthropometric indices used in this study could significantly serve as diagnostic indicators of metabolic syndrome at various cut-off points and accuracies (Table 3). The more accurate variables with their ability to predict metabolic syndrome defined by the NCEP ATP III criteria were triglyceride, BMI, and waist circumference (AUC=0.879, 0.811, and 0.874, respectively). Their respective cut-off, sensitivity, and specificity are represented in Table 3 as well as the confidence interval. Participants with triglyceride levels greater than 1.71 *μ*mol L1 were at a higher risk of being positive for metabolic syndrome as well as those with waist circumference >95 cm and BMI >24.8 kg m-2. Moreover, study participants with HDL-c less than or equal to 0.95 mmol L-1 were 100% specific for metabolic syndrome.

**Figure 3:**
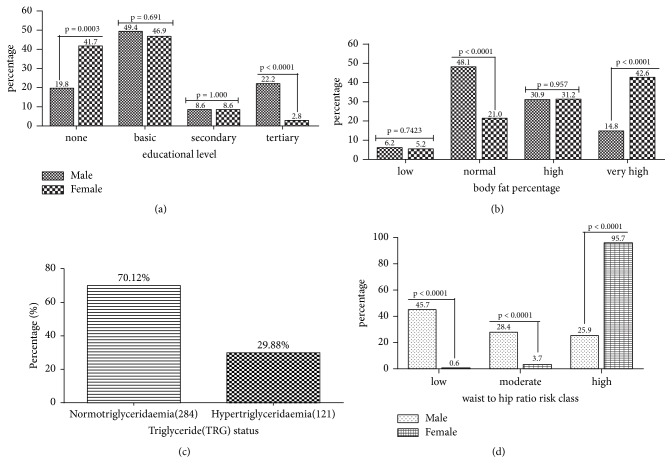
Percentage distribution of the study population by educational level (a), body fat percentage (b), triglyceride status (c), and waist-to-hip ratio (d).

Majority of the respondents had basic education, with the female showing a significantly higher level of no formal education than the males. The number of males that had obtained tertiary education at the time of the study was significantly higher than the females (Figure 3(a)). Greater body fat deposition was observed among the female participants with about seventy-three percent of them exhibiting high to very high body fat percentage (Figure 3(b)). Serum hypertriglyceridaemia was 29.88% among the study participants (Figure 3(c)). Most of the females enrolled for this study exhibited high central adiposity and hence fell within the high central obesity risk (WHR) group (Figure 3(d)).

Using the various cut-off point for the indices assessed as references in univariate multiple regression analysis to estimate the risk factor for metabolic syndrome among the study population, age above fifty-three (53) years and duration of disease greater than five (5) years were found not be significant predisposing factors for metabolic syndrome (Table 4). However, the female subjects had an approximately five (5) times risk of developing metabolic syndrome compared to their male counterparts. Among the biochemical diagnostic markers, an HbA1c level of greater than 5.43 percent increased the risk of metabolic syndrome approximately four (4) folds in the study subjects. Triglyceride levels above 1.71 mmol/L were the most indicative risk factor for a diabetic patient to develop metabolic syndrome. Thus subjects with this level of triglyceride had 82 times risk of presenting the condition compared to those with lower levels. The body's anthropometric characteristics of the patients were found to be significant risk determinants in the development of metabolic syndrome among the participants, with a risk index ranging four folds for waist-to-hip ratio greater than 0.958 to about twenty-four folds for waist circumference above 95 cm. (Table 4).

## 4. Discussion

The estimated mean age was 58.5 ± 9.9 years. Though the males were on average slightly younger than the females, the gender age difference was not statistically significant (p=0.554) (Table 1). In general, the anthropometric indices of the participants in this study were found to be significantly different in terms of gender, with the exception of waist-to-hip ratio which was found not to be significant. The females exhibited a higher mean waist circumference and average hip circumference as well as a higher body mass index (BMI) than males (28.3 ± 5.1, 26.5 ± 4.2 for female and male) (Table 2). Majority of the fat distribution markers were significantly higher among the female subjects in this study (Figure 1). It was observed from this study that female diabetic patients were more likely to present with abdominal obesity and reduced levels of high density lipoprotein cholesterol (HDL-c) than their male counterparts (Figure 1). Nevertheless, there were no statistical differences in raised fasting plasma glucose and triglyceride in relation to gender.

A relatively high prevalence of metabolic syndrome (MS) was observed among the study participants. A value of 90.6% of all participants in this study was classified as presenting with metabolic syndrome using the NCEP ATP III criteria (Figure 2). In earlier works of Titty et al. among type 2 diabetics at Komfo Anokye Teaching Hospital in [16] and Tamale Teaching Hospital in [17], with participants of similar characteristics like the current study, the prevalence of metabolic syndrome among the subjects reported was 55.9% and 60.3%, respectively. Though our documented prevalence of MS was higher compared to that reported by Titty et al., [16, 17], the probable reason may be because he and his colleagues recruited newly diagnosed diabetics of less than 1 year duration where complications such as hypertension and high abdominal circumference had not yet set in. However our results were quite similar to the observation of Kelliny et al., [18] who reported 80% of metabolic syndrome among diabetics in the Seychelles [18].

Gender differences in health vary according to differential vulnerabilities in men and women [19]. The susceptibility of female population to metabolic syndrome may be attributed to the interplay between sociodemographic factors and the corresponding biological outcomes [20]. The burden of metabolic syndrome was generally significantly higher among the female type 2 diabetics than their male counterparts (Figure 2; 94.43%, 76.54% for female and male respectively, p < 0.0001) in the current study. This is consistent with the reported gender difference in the prevalence of metabolic syndrome and its components among diabetics in the same setting by Titty, Owiredu [16] and Nsiah et al., [3]. Moreover, among other study sample populations and some in different geographical settings, significantly higher female sex difference in the prevalence of metabolic syndrome had also been documented, in Ghana [21–23], Kenya [24], in the Seychelles [18, 25], and in Russia.

Significantly higher number of the female participants presented very high body fat percentage (42.6%) compared to the males (14.8%), Figure 3(b). In many areas of sub-Sahara Africa, obesity constitutes an obvious social marker of poor knowledge and misconceptions about lifestyle risk factors which conflict with appropriate prevention and control measures. Furthermore, records of very high prevalence of obesity in urban women in Ghana have been reported by Danquah, Bedu-Addo [26]. According to (Wassink, Van Der Graaf [27, 28]) abdominal obesity is also strongly and independently associated with the incidence of type 2 diabetes. Excess amount and distribution of body adiposity (Figures 1 and 3(b)) have been associated with chronic metabolic diseases such as type 2 diabetes [29, 30], cardiovascular diseases [31, 32], and metabolic syndrome [33].

Serum hypertriglyceridaemia was 29.88% among the patients (Figure 3(c)). Results from a study in Spain confirm that the relationship between triglyceride levels and MS, independent of the presence of peripheral arterial disease, poses high risk for cardiovascular and other disease complications [34]. Though not too sensitive, high triglyceride levels were very specific and the strongest predictor for MS (AUC=0.879; specificity=97.4%) in our study (Table 3). As such, patients with triglyceride >1.71 mmol/l (p=0.001) were 82.1 times at risk for metabolic syndrome (Table 4). This may explain why the risk of myocardial infarction is high in metabolic syndrome [35].

Furthermore, the current study revealed significantly lower high waist-to-hip ratio risk among men to women (Figure 3(d)). Arthur et al. [36] identified waist circumference (WC) and waist-to-hip (WHR) but not body mass index (BMI) as significant predictors of metabolic syndrome in Ghanaian postmenopausal women [36]. The current study agreed partly with this finding in that BMI was also used in addition to WC and WHR to predict metabolic syndrome among type 2 diabetics in Ghana in the current study. Using the receiver operating characteristic (ROC) curve analysis, our finding showed similar but higher cut-off for WC (80.5 cm, versus 95 cm) and WHR (0.84 versus 0.96) compared to the earlier study of Arthur et al. [36], with both variables exhibiting strong prediction for MS (Table 3). The relatively high difference may be explained by the diabetic participants in this current study, and patients with waist circumference greater than 95 cm were 23.7 times at risk for MS (Table 4).

Educational level attainment has a strong positive effect on health behavior, attitudes, and, consequently, lifestyle choice. In the developing world, particularly Africa and for that matter Ghana disparities exist with gender in the level of educational attainment with higher level of education and older age, i.e., females drop out of school at an earlier age [22, 24, 37, 38]. Similarly, the observed gender difference in the distribution of metabolic syndrome in this study (Figure 3(a)) could also be casually attributed to differences in socioeconomic status, attainment of higher education, and the interplay of the two, since significantly more males (22.2%) had obtained tertiary education at the time of this study compared to 2.8% of females who also exhibited a significantly high proportion of participants without any formal education, Figure 3(a). Those with better education were likely to exercise more, eat healthier diets, etc., and this has been said to account for the sex difference in metabolic syndrome documented in Kenya by Kaduka [24].

## 5. Conclusions

The prevalence of metabolic syndrome observed among the study population was 90.6%, with females being likely to be affected than males. Additional risk factors which tend to increase the burden of MS in type 2 diabetes were found to be high plasma triglyceride and high body fat percentage, low educational status, and higher anthropometric measurements of waist circumference, hip circumference, waist-to-hip ratio, and BMI.

## Figures and Tables

**Table 1:** Baseline characteristics of the study population stratified by sex.

Variables	TOTAL	MALE	FEMALE	
	(n = 405)	(n = 81)	(n = 324)	*p-value*
Age (years)	58.5 ± 9.9	57.9 ± 10.4	58.6 ± 9.8	0.554
Duration of diabetes (years)	6.4 ± 5.4	6.1 ± 5.3	6.5 ± 5.5	0.518
FPG (mmol L^−1^)	9.3 ± 4.1	9.7 ± 5.2	9.1 ± 3.7	0.234
HbA1c (%)	7.1 ± 1.4	7.2 ± 1.4	7.1 ± 1.4	0.643
HbA1c >7%	198 (48.9)	42 (51.9)	156 (48.1)	0.551

Data are presented as mean ± SD. FPG: Fasting Plasma Glucose, HbA1c: glycated haemoglobin.

**Table 2:** Anthropometrics of the study population stratified by gender.

Variables	TOTAL	MALE	FEMALE	*p-value*
	(n = 405)	(n = 81)	(n = 324)	
Weight (kg)	70.2 ± 14.0	73.7 ± 12.5	69.3 ± 14.3	0.012
Height (m)	1.6 ± 0.1	1.7 ± 0.1	1.6 ± 0.1	< 0.001
BMI (kg/m^2^)	27.9 ± 5.0	26.5 ± 4.2	28.3 ± 5.1	0.004
WC (cm)	100.0 ± 12.2	98.3 ± 11.5	101.4 ± 12.3	0.040
HC (cm)	103.9 ± 9.7	101.2 ± 8.3	104.6 ± 9.9	0.005
WHR	1.0 ± 0.1	1.0 ± 0.1	1.0 ± 0.1	0.924

Data are presented as mean ± SD. BMI: body mass index; WC: waist circumference; HC: hip circumference; WHR: waist to hip ratio.

**Table 3:** Cut-off values of selected variables and their ability to predict metabolic syndrome defined by the NCEP-ATP III criteria.

Variable	Cut-off level	Sensitivity	95%CI	Specificity	95%CI	AUC
Age (years)	>53	70.3	65.3 - 74.9	39.5	24.0 - 56.6	0.504
Duration (years)	>5	44.1	39.0 - 49.4	68.4	51.3 - 82.5	0.562
FBG (mmol L^−1^)	>8.3	48.5	43.3 - 53.7	63.2	46.0 - 78.2	0.543
HbA1c (%)	>5.43	91.0	87.6 - 93.7	26.3	13.4 - 43.1	0.592
Triglyceride (*μ*mol L^−1^)	>1.71	68.9	63.9 - 73.6	97.4	86.2 - 99.9	0.879*∗*
HDL-c (mmol L^−1^)	≤0.95	26.4	22.0 - 31.3	100.0	90.7 - 100.0	0.671
BMI (kg m^−2^)	>24.8	76.0	71.3 - 80.3	71.1	54.1 - 84.6	0.811*∗*
WC (cm)	>95	73.6	68.7 - 78.0	89.5	75.2 - 97.1	0.874*∗*
HC (cm)	>101	58.9	53.6 - 63.9	86.8	71.9 - 95.6	0.777
WHR	>0.958	59.1	53.9 - 64.2	76.3	59.8 - 88.6	0.713

HbA1c: glycated haemoglobin; FPG: Fasting Plasma Glucose; BMI: body mass index; WC: waist circumference; HC: hip circumference; WHR: waist to hip ratio; HDL-c: high density lipoprotein cholesterol. Almost all variables were likely to predict MS, with the main being triglyceride*∗*, Waist Circumference*∗* and BMI*∗*.

**Table 4:** Univariate analysis of the study variables as risk factors for metabolic syndrome in diabetic patients.

Variables (*response*)	OR(95%CI)	p-value
Age (*>53 years*)	1.5(0.8 - 2.1)	0.216
Duration (*>5 years*)	1.7(0.8 - 3.5)	0.132
Sex (f*emale*)	4.9(2.5 - 9.8)	0.001
FPG (*>8.3 mmol L*^*-1*^)	1.6(0.8 - 3.2)	0.174
HBA1c (*>5.43 %*)	3.6(1.6 - 8.1)	0.002
Triglyceride (*>1.71 mmol L*^*-1*^)	82.1(11.1 - 605.9)	0.001
BMI (*>24.8 kg m*^*-2*^)	7.7(3.7 - 16.1)	0.001
Waist circumference (*>95 cm*)	23.7(8.2 - 68.4)	0.001
Hip circumference (*>101 cm*)	9.4(3.6 - 24.7)	0.001
Waist to hip ratio (*>0.958*)	4.1(1.9 - 8.7)	0.001
Body fat percentage (*>32.6*)	14.2(5.8 - 35.1)	0.001

There was a significant relationship between variables and MS with the exception of age, duration of diabetes, and Fasting Plasma Glucose (FPG).

## Data Availability

The data used to support the findings of this study are available from the corresponding author upon request.

## Abbreviations

MS/MetS: Metabolic Syndrome
FPG: Fasting Plasma Glucose
HbA1c: Glycated haemoglobin
BMI: Body Mass Index
NCEP ATP III: National Cholesterol Education Program Adult Treatment Panel III
HDL-c: High Density Lipoprotein Cholesterol
LDL-c: Low Density Lipoprotein Cholesterol
WC: Waist Circumference
HC: Hip Circumference
WHR: Waist-to-Hip Ratio
TG/TRG: Triglyceride.
